# A Case of Insanity Cured by Chloroform

**Published:** 1851-07

**Authors:** Asahel Clarke

**Affiliations:** Beloit, Rock Co., Wis.


ARTICLE III.

*A case of Insanity cured by Chloroform.*—By ASAHEL CLARKE, M.D., of Beloit, Rock Co., Wis.

On the evening of the 19th of March last, I was called to see Mrs. F., aged 49. For two or three months previous to this time, she had been gloomy and desponding. During the day a letter had been received from California, containing unpleasant intelligence. Several hysterical convulsions followed. She had recovered from these when I arrived, but appeared to be insane. I could not persuade her to take any medicine, and left, advising her attendants, if she did not get sleep during the night, and was not in her right mind in the morning, to send for her husband. (Mr. F. had gone to the County Seat as a juryman.)

He was sent for, and the family physician also, (a Homœopathist.) Ten days after I was requested to see her again.

She has had but little sleep, and has continued to be entirely insane ever since my last visit. I advised the use of chloroform. Mr. F., after making many objections, consented to have it given. I gave it till it produced quiet sleep, and left, giving directions to have the room kept still till she awoke.—She slept about twenty minutes after I left her, and awoke quite rational, and has continued so ever since. After she awoke, she commenced talking about matters that occurred during the day of my first visit. The ten days that had elapsed since, were wholly lost time to her. She has no recollection of any thing that occurred during that time.

If you think the above, or any part of it, would be of interest to your readers, you are at liberty to publish it.

*April* 23, 1851.

